# An Advanced Multiplex Real-Time Reverse Transcription Loop-Mediated Isothermal Amplification Assay for Rapid and Reliable Detection of Porcine Epidemic Diarrhea Virus and Porcine Internal Positive Control

**DOI:** 10.3390/v15112204

**Published:** 2023-11-01

**Authors:** Hye-Ryung Kim, Jong-Min Kim, Ji-Su Baek, Jonghyun Park, Won-Il Kim, Bok Kyung Ku, Hye-Young Jeoung, Kyoung-Ki Lee, Choi-Kyu Park

**Affiliations:** 1 College of Veterinary Medicine & Institute for Veterinary Biomedical Science, Kyungpook National University, Daegu 41566, Republic of Korea; gpfuddl25@knu.ac.kr (H.-R.K.); kjm51062@knu.ac.kr (J.-M.K.); sy20103712@knu.ac.kr (J.-S.B.); parkjh@knu.ac.kr (J.P.); 2 College of Veterinary Medicine, Jeonbuk National University, Iksan 54596, Republic of Korea; kwi0621@jbnu.ac.kr; 3 Animal and Plant Quarantine Agency, Gyeongsangbuk-do, Gimcheon 39660, Republic of Korea; kubk@korea.kr (B.K.K.); jhy98@korea.kr (H.-Y.J.); naturelkk@korea.kr (K.-K.L.)

**Keywords:** PEDV, multiplex real-time RT-LAMP, assimilating probe, N gene, *Sus scrofa β-actin* gene, internal positive control

## Abstract

For rapid and reliable detection of porcine epidemic diarrhea virus (PEDV) from pig clinical samples, a multiplex, real-time, reverse transcription loop-mediated isothermal amplification (mqRT-LAMP) was developed using two sets of primers and assimilating probes specific to the PEDV N gene and the *Sus scrofa β-actin* gene, which was used as an endogenous internal positive control (EIPC) to avoid false-negative results. The assay specifically amplified both target genes of PEDV and EIPC in a single reaction without any interference but did not amplify other porcine viral nucleic acids. The limit of detection was 10 copies/μL, 100-fold lower than that of a reverse transcription-polymerase chain reaction (RT-PCR) and equivalent to that of quantitative/real-time RT-PCR (qRT-PCR). This assay has high repeatability and reproducibility with coefficients of variation < 4.0%. The positive signal of the mqRT-LAMP assay was generated within 25 min, demonstrating advantages in rapid detection of PEDV over RT-PCR or qRT-PCR assay, which require at least 2 h turnaround times. In clinical evaluation, the detection rate of PEDV by mqRT-LAMP assay (77.3%) was higher than that of RT-PCR assay (69.7%), and comparable to qRT-PCR (76.8%) with almost 100% concordance (kappa value 0.98). The developed mqRT-LAMP assay can serve as an advanced alternative method for PEDV diagnosis because it has high sensitivity and specificity, rapidity, and reliability even in resource-limited laboratories.

## 1. Introduction

The porcine epidemic diarrhea virus (PEDV), a member of the *Alphacoronavirus* genus in the *Coronaviridae* family, is an enveloped, single-stranded, positive-sense RNA virus that causes severe swine enteric disease characterized by vomiting, watery diarrhea, dehydration, and high mortality in suckling piglets, resulting in significant economic losses in the swine industry [1,2]. The PEDV genome is approximately 28 kb in length and composed of a 5′ untranslated region (UTR), at least seven open reading frames (ORF1a, ORF1b, and ORFs 2–6), and a 3′ UTR. ORF 1a and 1b encode replicase polyprotein (pp) la and pp1ab, respectively, and ORFs 2–6 encode four structural proteins consisting of the spike (S), envelope (E), membrane (M), and nucleocapsid (N) proteins; ORF3 encodes an accessory protein [3].

PEDV infection was initially identified in the early 1970s in Europe. Since then, the disease has been reported in various European and Asian countries. However, the most severe epidemics have predominantly occurred in Asian countries including Japan, China, the Republic of Korea, the Philippines, Thailand, Taiwan, and Vietnam [3]. A highly pathogenic PEDV was unexpectedly detected in the United States in April 2013. Since then, the virus has rapidly spread across the country as well as Canada and Mexico, resulting in the death of more than eight million newborn piglets in the US alone during the one-year suffering of the epidemic [4,5,6,7,8,9]. Moreover, sudden severe PED outbreaks have become more frequent in Asian countries, such as the Republic of Korea, Taiwan, and Japan, even on pig farms that are vaccinated with commercial PEDV vaccines [9,10,11]. Consequently, PED is now being recognized as a serious emerging, reemerging, and rapidly spreading global disease that causes large economic losses to the pig industry worldwide [12].

The rapid and reliable detection of PEDV is crucial to control measures in a timely manner and prevent the further spread of the virus. Currently, molecular diagnoses such as reverse transcription-polymerase chain reaction (RT-PCR) and quantitative/real-time RT-PCR (qRT-PCR) are widely used for the detection of PEDV [13,14,15,16,17]. However, these methods are limited by their complicated procedures for the detection of amplified products, cost-effectiveness, and the requirement for sophisticated equipment and specialized labor. This makes them unsuitable for on–the–spot detection in field situations or in sparsely equipped laboratories in developing countries. The development of a sensitive, specific, rapid, and cost–effective assay usable both in the laboratory and on-site is imperative to detect PEDV in suspected cases. One such promising method is the loop-mediated isothermal amplification (LAMP) assay, which is considered a valuable tool for detecting various pathogens due to its high sensitivity, specificity, rapidity, and simplicity [18,19,20].

To date, several reverse transcription-LAMP (RT-LAMP) assays have been developed for the detection of PEDV [21,22,23,24,25,26]. Previously reported RT-LAMP assays for PEDV determined the assay results by gel electrophoresis, turbidity analysis using a real–time turbidimeter, dye-mediated DNA intercalating (Pico-green or SYBR Green 1 or SYTO 9) or metal colorimetric indicator-mediated (hydroxynaphthol blue) visual detection, or a vertical flow visualization strip [21,22,23,24,25,26]. However, these are non-specific and indirect indicators for confirming amplified DNA, and their results may be difficult to interpret when the amount of template in the tested sample is low. In an effort to overcome the drawbacks of these relatively non-specific monitoring methods, several attempts have been made to develop a probe-based detection system for LAMP. These include systems that detect amplification by incorporating quenching probes [27], assimilating probes [28], molecular beacons [29], loop primers for self-de-quenching-LAMP [30], and bi-labelled backward loop [31]. Each of these gene-specific detection systems was able to enhance the sensitivity, specificity, and quantification capability of conventional LAMP assay. Furthermore, these assays can be designed to include multiple probes with different fluorophores, enabling the simultaneous detection of multiple target sequences in a single reaction. However, there are currently no RT-LAMP assays utilizing these probe-based detection systems that are capable of real-time detection of PEDV. Therefore, we developed a probe-based real-time RT-LAMP (qRT-LAMP) assay to address this. To monitor potential problems that may occur throughout the reaction, such as issues arising during sample collection, transport and storage, and nucleic acid extraction, and from the presence of reaction inhibitors in clinical samples, we also included a set of primers and probes targeting the β-actin gene of *Sus scrofa* as an EIPC in the PEDV qRT-LAMP assay. In this study, we developed probe-based multiplex qRT-LAMP (mqRT-LAMP) assay combined with EIPC is intended to yield higher sensitivity, rapidity, and reliability by using a target gene-specific assimilating probe that facilitates rapid and specific detection from clinical pig samples.

## 2. Materials and Methods

### 2.1. Samples and Nucleic Acid Extraction

A KNU141112-S DEL5/ORF3 vaccine strain (GenBank accession number KY825243) was used to develop and optimize the mqRT-LAMP conditions. Other porcine viruses were obtained from the Animal and Plant Quarantine Agency (Gimcheon, Republic of Korea), or Institute for Veterinary Biomedical Science, Kyungpook National University (Daegu, Republic of Korea) and used for the evaluation of the assay’s specificity. These consisted of transmissible gastroenteritis virus (TGEV, strain 175Lvac), porcine deltacoronavirus (PDCoV, strain KNU16-07), porcine rotavirus (PRV, strain A1va), type 1 porcine reproductive and respiratory syndrome virus (PRRSV, strain Lelystad virus), type 2 PRRSV (strain LMY), classical swine fever virus (CSFV, strain LOM), porcine circovirus 2 (PCV2, strain PCK0201), porcine parvovirus (PPV, strain NADL-2), swine influenza virus (SIV, strain VDS1), and Aujeszky’s disease virus (ADV, strain YS). For clinical evaluation of the mqRT-LAMP assay, a total of 185 PED-suspected clinical samples (69 fecal and 116 intestinal samples) were collected from Korean pig farms where animals showed clinical signs of diarrhea. Total nucleic acid was extracted from 200 μL of a virus stock and field samples using a TANBead Nucleic Acid Extraction Kit with a fully automated magnetic bead operating platform (Taiwan Advanced Nanotech Inc., Taoyuan, Taiwan) according to the manufacturer’s instructions. All RNA and DNA samples were allocated and stored at −80 °C until use.

### 2.2. Construction of PEDV Reference Gene for mqRT-LAMP Assay

The *N* gene of the KNU141112-S DEL5/ORF3 vaccine strain was amplified by RT-PCR using primers that include the forward primer 5′- TTATGGCTTCTGTCAGTTTC-3′ and the reverse primer 5′-ACATTGTTTAATTTCCTGTGTC-3′, as previously described [26]. RT-PCR was performed using a commercial RT-PCR kit (Inclone™ one-step RT-PCR kit, Inclone Biotech, Seongnam, Republic of Korea). The 25 μL reaction mixture, which contained 12.5 μL of 2 × reaction buffer, 0.4 μM of each primer, and 5 μL of PEDV RNA as a template, was prepared according to the manufacturer’s instructions. The amplification was carried out in a thermal cycler (Applied Biosystems, Foster City, CA, USA) under the following conditions: reverse transcription at 45 °C for 50 min, initial denaturation at 95 °C for 15 min, followed by 35 cycles at 95 °C for 20 s, 55 °C for 40 s, and 72 °C for 3 min, and a final extension at 72 °C for 5 min. The amplified 1326 bp N gene was purified and cloned into the pTOP TA V2 vector using the TOPclone TA core kit (Enzynomics, Daejeon, Republic of Korea). The recombinant plasmid DNA samples were linearized by EcoRI and purified using the Expin CleanUP SV kit (GeneALL Biotechnology, Seoul, Republic of Korea). Subsequently, in vitro RNA transcription was performed using a RiboMAX Large Scale RNA Production System-T7 (Promega, Madison, WI, USA) according to the manufacturer’s instructions. RNA concentrations were determined by measuring the absorbance at 260 nm using a NanoDrop Lite spectrophotometer (Thermo Fisher Scientific, Waltham, MA, USA). RNA transcript copy numbers were quantified using a previously described method [32]. Ten-fold dilutions of the RNA sample (from 10^6^ to 1 copies/μL) were stored at −80 °C and used as a standard RNA of the PED N gene.

### 2.3. RT-PCR and qRT-PCR Assay

RT-PCR was performed as previously described [25] with primers (P1 and P2) specific for the PEDV N gene using a commercial RT-PCR kit (PrimeScript™ one-step RT-PCR kit, Takara, Seoul, Republic of Korea) in a 50 μL reaction mixture containing 25 μL of 2 × buffer, 2 μL of enzyme mix, 0.4 μM of each primer, 5 μL of the RNA template, and dH_2_O to reach the final volume, according to the manufacturer’s instructions. Amplification was conducted using a thermal cycler (Applied Biosystems), and the following PCR protocol was used: reverse transcription at 50 °C for 30 min, initial denaturation at 94 °C for 2 min, followed by 35 cycles of amplification (94 °C for 30 s, 55 °C for 30 s, and 72 °C for 40 s), and a final extension at 72 °C for 2 min. The expected 428 bp amplicons were visualized using 1.5% agarose gel electrophoresis and staining with a NEO green dye (Neoscience, Suwon, Republic of Korea).

The qRT-PCR assay for PED was performed with N gene-specific primers and probes according to previously described methods [13]. The primers and probe sequences of qRT-PCR were modified to reflect the target gene sequence variation of currently circulating PEDV strains [26]. The details of the primers and probe used in the qRT-PCR assays are provided in Table 1. qRT-PCR was performed using a commercial one-step RT-PCR kit (Toyobo Co., Ltd., Osaka, Japan) in a 20 μL reaction mixture containing 0.5 μL of DNA polymerase, 0.5 μL of RT enzyme mix, 10 μL of 2 × reaction buffer, 0.4 μM of each PEDV primer and probe, 5 μL of RNA, and 1 μL of water. The reaction was carried out in a CFX96 Touch™ Real-Time PCR Detection System (Bio-Rad, Hercules, CA, USA). The reaction was performed under the following conditions: initial reverse transcription at 50 °C for 10 min, initial denaturation at 95 °C for 1 min, 40 cycles of denaturation at 95 °C for 15 s, and annealing and extension at 58 °C for 45 s. Real-time fluorescence values of the FAM-labeled probe were measured in ongoing reactions at the end of each annealing step. To interpret the qRT-PCR results, samples producing a cycle threshold (Ct) of <37 were considered positive, whereas those with a higher Ct value were considered negative based on the limit of detection (LOD) of the assay.

### 2.4. Primers and Assimilating Probes for PEDV N Gene and Internal Positive Control

For the detection of PEDV *N* and *Sus scrofa β-actin*, two sets of primers and probes were used for the mqRT-LAMP assay. Six basic LAMP primers consisting of two outer primers (F3 and B3), two inner primers (FIP and BIP), and two loop primers (LF and LB) for detecting the PED N gene were used as previously reported by our research teams [26]. In the present study, six basic LAMP primers and assimilating probes to detect the *Sus scrofa β-actin* gene and assimilating probe for PEDV N gene detection were newly designed using Primer Explorer V5 software (Fujitsu System Solutions, Ltd., Tokyo, Japan). To monitor LAMP reactions for PEDV *N* and *Sus scrofa β-actin* in real time, the target gene-specific assimilating probe pairs were designed featuring fluorescent strands (F strand) and quencher strands (Q strand) (Table 1). Assimilating probe pairs were designed based on a previous report [28,33]. The sequence to assimilate the probe pairs was randomly selected to ensure that there were no non-specific reactions in the target species, and the probes were carefully designed to prevent the creation of secondary structures for all primers and probes including the mqRT-LAMP assay. The F strand has an assimilating sequence that is added and elongated to the 5′ end of the LF primer sequence, and the Q strand is a complementary sequence of the added assimilating sequence in the F strand. The F strand for the PEDV N gene was designed using the LF primer region and labeled with FAM (6-carboxy-fluorescein) at the 5′ end, and the Q strand was labeled with Black Hole Quencher 1 (BHQ1) at the 3′ end. For simultaneous and differential detection of the PEDV N and *Sus scrofa β-actin* genes, it is essential that the sequence-specific probes are labeled with reporter dyes whose fluorescence spectra are distinct or exhibit minimal overlap [34]. The F strand for detecting the *Sus scrofa β-actin* gene in this study was designed using the LF primer region and labeled with HEX (6-carboxy-2′,4,4′,5′,7,7′-hexachlorofluorescein) at the 5′ end, and the Q strand was labeled with BHQ1 at the 3′ end. The details of all primers and probes are provided in Table 1. The OligoAnalyzer™ Tool (Integrated DNA Technologies, Coralville, IA, USA; https://sg.idtdna.com/pages/tools/oligoanalyzer, last accessed on 15 January 2023) was used to check the formation of the secondary structure that might interfere with the LAMP amplification. The specificity of the primers and probes for the mqRT-LAMP was confirmed against random nucleotide sequences using a BLAST search of the NCBI GenBank database (http://www.ncbi.nlm.nih.gov/BLAST/, last accessed on 15 January 2023). All primers and probes were synthesized using BIONICS (Seoul, Republic of Korea).

### 2.5. Optimization of mqRT-LAMP Assay

Before establishing the mqRT-LAMP assay, reaction conditions for the monoplex qRT-LAMP assay of PEDV were optimized. The crucial first step of monoplex qRT-LAMP assay using the assimilating probe is the ratio of F and Q strands in the reaction mixture [28,33,35,36]. To determine the optimal concentration, monoplex qRT-LAMP reactions were tested by changing the concentration of the Q strand (0.08, 0.12, 0.16, 0.24, 0.32 μM) after fixing the concentration of the PEDV-*N*-F strand (0.08 μM), and the remaining concentrations were maintained at the composition ratio of RT-LAMP used in our previous report [26]. All qRT-LAMP assay-related experiments including optimization were performed using a commercial kit (Mmiso^®^ RNA amplification kit (real-time), M-monitor Inc., Daegu, Republic of Korea). The reaction mixture contained 2 × reaction buffer, enzyme mix, 1.6 μM inner primers (FIP and BIP), 0.2 μM outer primers (F3 and B3), 0.8 μM loop primers (LF and LB), 5 μL of template RNA and dH_2_O to bring the final volume to 25 μL. mqRT-LAMP was carried out with 10^3^ copies/μL of RNA transcript from the PEDV N gene of the KNU141112-S DEL5/ORF3 strain described above. After optimization of the monoplex qRT-LAMP assay, mqRT-LAMP assays were adjusted. First, the concentration ratio of the two sets of primers and the probe were optimized, followed by adjustment of the concentration of *Bst* polymerase (8 U, 12 U, 16 U, 18 U). Other reaction components were the same as those used for the monoplex qRT-LAMP assay. mqRT-LAMP was also carried out at different reaction temperatures (57–62 °C in 1 °C increments) to determine the optimal reaction temperature while keeping the reaction time fixed at 40 min. Real-time fluorescence signals of a FAM-labelled assimilating probe PEDV F strand and a HEX-labelled assimilating probe EIPC F strand were measured at 60 s intervals during the reactions. The time to positive (Tp) values for each sample was determined as previously described [28]. All experiments were carried out in triplicate to ensure reproducibility and repeatability.

### 2.6. Specificity and Sensitivity of mqRT-LAMP Assay

To test the specificity of the mqRT-LAMP assay, the assay was performed using RNA templates extracted from four genogroups of PEDV isolates (KNU1305, SM98, DR13, and CV777), ten control virus samples (PDCoV, PRV, type 1 and 2 PRRSVs, TGEV, CSFV, PCV2, PPV, SIV, and ADV), and uninfected cell cultures (PK-15 cells and Vero cells) as negative controls. The limit of detection (LOD) of the mqRT-LAMP assay was determined using 10-fold serial dilutions of a PEDV N gene standard RNA, ranging from 10^6^ to 10^0^ copies/μL. Subsequently, the LOD of the mqRT-LAMP assay was compared with those of the RT-PCR and qRT-PCR assays using the RNA templates described above. The detection software also calculated the correlation coefficient (*R*^2^) of the standard curve, the standard deviations of the results, and PED RNA copy numbers of the samples based on the standard curve. The efficiency of the assay was determined using previously described calculations [37]. To confirm the interference in amplification and detection between target PEDV RNA and EIPC, the mqRT-LAMP assay was performed with nucleic acids extracted by spiking PEDV N gene standard RNA (10-fold dilutions) into a negative intestinal and fecal sample that was not infected with any other swine viruses.

### 2.7. Precision of mqRT-LAMP

Following MIQE guidelines [37], three different dilutions of PEDV N gene standard RNAs (10^6^, 10^4^, and 10^2^ copies/μL) were used to determine repeatability (intra-assay precision) and reproducibility (inter-assay precision). For intra-assay variability, the results from each diluted sample were obtained from three separate experiments performed on the same day. For inter-assay variability, each diluted sample was tested in six independent experiments performed by two different operators on different days. The coefficient of variation (CV) for the Tp values was determined based on the intra-assay or inter-assay results and expressed as a percentage of the mean value together with the standard deviation.

### 2.8. Clinical Evaluation of mqRT-LAMP

For clinical evaluation of the mqRT-LAMP assay, 185 clinical pig samples (69 fecal and 116 intestinal samples) were then tested using the developed mqRT-LAMP assay, and the results were compared with the results of RT-PCR and qRT-PCR. The concordance between mqRT-LAMP and RT-PCR or qRT-PCR results was analyzed using Cohen’s kappa statistics at a 95% confidence interval (CI) [38]. The interpretation of the calculated kappa coefficient value (κ) was as follows: κ < 0.20, slight agreement; 0.21–0.40, fair agreement; 0.41–0.60, moderate agreement; 0.61–0.80, substantial agreement; and 0.81–1.0, almost perfect agreement. Pearson’s correlation coefficient (r) was used to interpret the strength of the relationship between the Tp value of mqRT-LAMP and the Ct value of qRT-PCR for PEDV-positive clinical samples. The correlation between both assays was considered to be highly positive when the calculated r value was in the range of 0.71–1.0 [39]. For Pearson’s correlation coefficient and linear regression analysis and data visualization, PyCharm 2023.1.3 software (Professional Edition; JetBrains, Praha, Czech Republic) was used, including commonly available free packages (numpy, seaborn, pandas, sklearn-linear, matplotlib). To compare the diagnostic speed for clinical evaluation results of the two assays, the Ct value of qRT-PCR was converted to reaction times in minutes. Since the fluorescence signal of mqRT-LAMP was originally set to monitor at 1 min intervals, the turnaround time of mqRT-LAMP for the detection of PEDV RNA from each sample was determined considering a Tp value of 1 as 1 min. In the case of qRT-PCR, the reaction processes are subdivided into reverse transcription, initial denaturation, and PCR cycling steps including denaturation, annealing, and extension stages. Recording the turnaround time for each reaction step using our real-time detection system yielded respective durations of 10, 1, and 77 min for these steps. Therefore, the total reaction time (time elapsed) for qRT-PCR was calculated by multiplying the Ct value by 1.9 min and adding 11 min.

## 3. Results

### 3.1. Optimization of the mqRT-LAMP Assay

**Figure 1:**
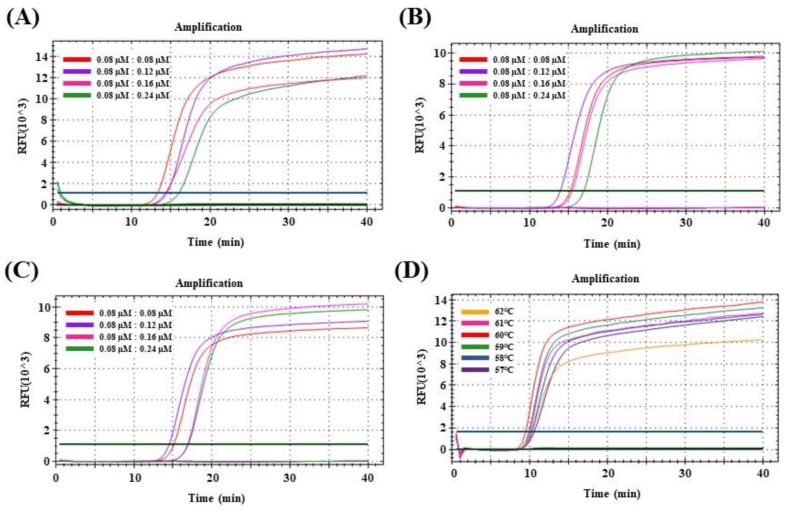
Optimization of monoplex and multiplex real-time reverse transcription loop-mediated isothermal amplification (qRT-LAMP) conditions for detection of porcine epidemic diarrhea virus (PEDV). The results of fluorescence detection show fluorescence strand (F strand) and quench strand (Q strand) ratios of 1:1 (0.08 µM:0.08 µM), 1:1.5 (0.08 µM:0.12 µM), 1:2 (0.08 µM:0.16 µM), 1:3 (0.08 µM:0.24 µM), and 1:4 (0.08 µM:0.32 µM). Each figure represents the results of monoplex and multiplex qRT-LAMP assay conducted on 10^3^ copies/μL PEDV N gene standard RNAs. (**A**) Result of the fluorescence-optimized test of monoplex qRT-LAMP using 10^3^ copies/μL of PEDV N gene standard RNA. (**B**) Result of the fluorescence-optimized test of multiplex qRT-LAMP for PEDV N gene detection combined with pig whole nucleic acid extracted from intestines. (**C**) As (**B**), extracted from feces. (**D**) Fluorescence signals of the multiplex qRT-LAMP assay at different reaction temperatures (57–62 °C).

Using the monoplex or multiplex qRT-LAMP assay with PEDV RNA templates or the templates including the *Sus scrofa β-actin* gene, a positive fluorescence signal was detected in real-time that was directly dependent on the concentration ratios of the F and Q strands. When the concentration ratio of monoplex PEDV F and Q strands was adjusted to 1:1 (0.08 μM:0.08 μM), the lowest Tp values were obtained for PEDV-standard RNA (Figure 1A). The lowest Tp values of multiplex qRT-LAMP were confirmed for PEDV-standard RNA containing the pig whole nucleic acid extracted from the intestine and feces when the concentration ratio of the F and Q strands was adjusted to 1:1.5 (0.08 μM:0.12 μM) (Figure 1B,C). When comparing by increasing the concentration of Q strands used in the two common sets, the concentration of the common Q strand was optimized to 0.12 μM. The reaction conditions of mqRT-LAMP were adjusted based on these values, with concentrations of the PEDV and EIPC primer sets adjusted to 1.6 μM inner primer (FIP and BIP), 0.2 μM outer primers (F3 and B3), 0.8 μM loop primers (LF and LB), and 0.08 μM F strand. Finally, the concentration of the enzyme was increased and evaluated, and optimal reaction conditions were determined with 12 U of *Bst* polymerase. The lowest Tp value of the mqRT-LAMP assay was obtained when the reaction temperature was 60 °C for PEDV N gene standard RNAs (Figure 1D).

### 3.2. Specificity of the mqRT-LAMP Assay

To test the specificity of the mqRT-LAMP assay, the PEDV strain (KNU141112 -S DEL5/ORF3), ten other porcine viruses (TGEV, PDCoV, PRV, type 1 and 2 PRRSVs, CSFV, PCV2, PPV, SIV, ADV), and two cell culture (Vero cells and PK-15 cells) were tested using mqRT-LAMP. This yielded PED-positive results for the PEDV strain only and negative results for all other porcine pathogens, cell cultures, and negative controls. The assay yielded EIPC-positive results for PK-15 cells which originated from pig kidney cells as well as for virus isolates that contained porcine cellular materials, whereas the EIPC signal did not detect Vero cells that originated from African green monkeys (Table 2). In addition, EPIC-positive HEX signals were not detected in any of the five pathogen strains that were not cultured in pig-derived cell lines. Thus, it can be concluded that the primer set used for this assay shows a high degree of specificity for the PEDV N and *Sus scrofa β-actin* genes.

### 3.3. Sensitivity Comparison of mqRT-LAMP with RT-PCR and qRT-PCR Assay

**Figure 2:**
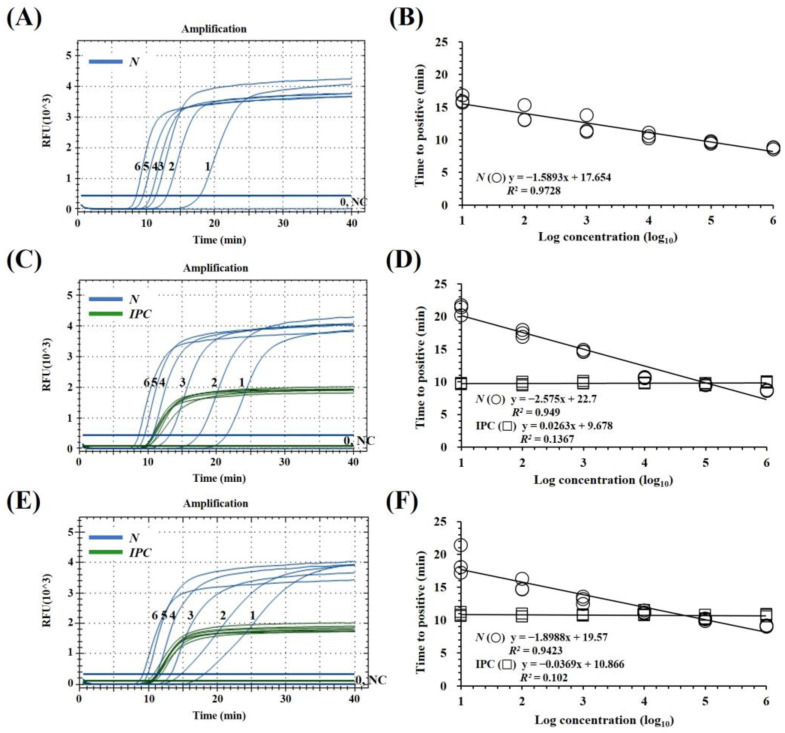
Sensitivities of multiplex real-time reverse transcription loop-mediated isothermal amplification (mqRT-LAMP) assay in the presence or absence of pig samples of intestine and feces, respectively. (**A**,**B**) Limit of detection and standard curve of mqRT-LAMP assay with only PEDV N gene standard RNA template. (**C**,**D**) As (**A**,**B**) for PEDV N gene standard RNAs spiked into virus-free pig samples of intestine. (**E**,**F**) As (**C**,**D**) for samples of feces. Amplification curves are shown for one of three replicates. Lines 6–0 show a 10-fold serial dilution of RNAs (from 10^6^ to 1 copies/μL). NC, negative controls (nuclease-free water). Serial 10-fold dilutions of each PRRSVs viral RNA standard (from 10^6^ to 1 copies/µL) were plotted against time to positive (Tp). The coefficient of determination (*R*^2^) and equation of the regression curve (y) were calculated using CFX Maestro Software (Bio-Rad).

To determine the LODs of the mqRT-LAMP assay and to compare the LODs of RT-PCR and qRT-PCR assays, the assays were performed using PEDV N gene standard RNA dilutions ranging from 10^6^ to 1 copies/μL. The LOD of mqRT-LAMP (10 copies/μL) was comparable to monoplex qRT-LAMP (10 copies/μL) and qRT-PCR (10 copies/μL) and 100-fold lower than those of RT-PCR (10^3^ copies/μL) (Supplementary Figure S1). For a more detailed analysis, the LODs of the mqRT-LAMP and qRT-PCR assays were determined using triplicate experiments with 50, 40, 30, 20, 10, and 5 copies of PEDV N gene standard RNA per reaction. The LOD was determined as the lowest dilution factor at which positive reactions were obtained in all three replicates. Finally, a mean detection limit of approximately 40 copies (corresponding to a Ct value of 37) per reaction was established for the mqRT-LAMP and qRT-PCR assay. In addition, to evaluate the diagnostic performance of the mqRT-LAMP assay, the 10-fold dilutions of PED N gene-standard RNA (10^6^–10^0^ copies/μL) were spiked into pig intestinal and fecal samples that were not infected with PEDV or any other swine viruses. It was confirmed that EIPC LAMP primers/probes were consistently detected for each sample type regardless of the concentration of PEDV N gene standard RNAs. To determine the linearity of the assay, standard curves for the target genes were generated by plotting the Tp value versus their dilution factors. The correlation coefficient (*R*^2^) of the mqRT-LAMP assay was 0.972 over the total concentration range when only PEDV N gene standard RNAs were present in the reaction solution, but 0.949 or 0.942 when nucleic acid from spiking PEDV N gene standard RNAs into intestine or feces was used. In contrast, Tp values were highly correlated with copy number over the range of 10^6^–10^4^ copies/μL (*R*^2^ > 0.98). This result suggests that mqRT-LAMP assay is semi-quantitative (Figure 2).

### 3.4. Precision of mqRT-LAMP

Intra-assay repeatability and inter-assay reproducibility were assessed with two distinct operators on different days using three different concentrations (high, medium, and low) of each PEDV N gene standard RNA and were tested in triplicate in six different runs. For the N gene, the coefficients of variation within runs (intra-assay variability) ranged from 2.65% to 3.76%; in contrast, inter-assay variability ranged from 0.95% to 1.74% (Table 3). These results demonstrate that the developed mqRT-LAMP method is accurate and reliable for detecting PEDV.

### 3.5. Clinical Evaluation of mqRT-LAMP

A clinical test was conducted with 185 clinical pig samples (69 fecal samples and 116 intestinal samples). EIPC signals were generated using the developed mqRT-LAMP assay in all but eight tested clinical samples (five fecal and three intestinal samples), indicating that porcine cellular material was not included or degraded in the clinical samples. The total detection rates of mqRT-LAMP, RT-PCR, and qRT-PCR were 77.3%, 76.8%, and 69.7%, respectively (Table 4). For intestinal samples, the detection rate of PEDV RNA was 45.9% (85/185) for the mqRT-LAMP and qRT-PCR assays, and 42.7% (79/185) for the RT-PCR assay. For fecal samples, it was 31.4% (58/185) for the mqRT-LAMP assay, and 30.8% (57/185) and 27.0% (50/185) for the qRT-PCR and RT-PCR assays, respectively. The rates of positive, negative, and overall agreements for the mqRT-LAMP assay relative to the qRT-PCR and RT-PCR assays were 99.3% (142/143), 100% (42/42), and 99.5% (184/185), and 90.2% (129/143), 100% (42/42), and 92.4% (171/185), respectively.

The mqRT-LAMP assay yielded a PEDV-N gene-positive result for one additional fecal sample compared to the qRT-PCR assay. Additionally, when compared to the RT-PCR assay, the mqRT-LAMP assay identified a further eight positive fecal samples and six positive intestinal samples. All discordant samples were confirmed to have positive signals for EIPC (Supplemental Table S1) and were analyzed with the DNA sequence of the mqRT-LAMP amplicons using F3 and B3 primers of mqRT-LAMP with Sanger sequencing by a commercial company (BIONICS). The 191 bp fragment sequences of all discordant samples were identified as having 100% similarity for the PEDV N gene using BLAST. This indicates that the mRT-LAMP response is more sensitive to clinical samples. The causes of the result determined for the single discordant fecal sample remain unclear; this may be due to the differences in sensitivity between each assay or the presence of unknown reaction inhibitors in the fecal samples. The kappa value (95% CI) was 0.80 (0.71–0.91) between mqRT-LAMP and RT-PCR, and 0.98 (0.95–1.02) between mqRT-LAMP and qRT-PCR. These results indicate substantial agreement between the diagnostic results of mqRT-LAMP and RT-PCR, and almost perfect agreement between mqRT-LAMP and qRT-PCR.

### 3.6. Comparative Analysis of Reaction Speed According to Clinical Evaluation Results of mqRT-LAMP and qRT-PCR

**Figure 3:**
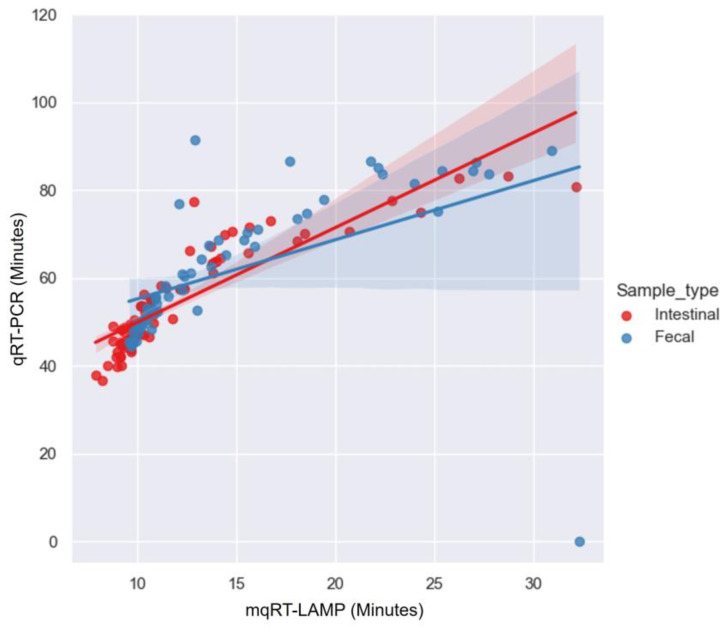
Scatter plot of the clinical evaluation results of multiplex real-time reverse transcription loop-mediated isothermal amplification (mqRT-LAMP) and real-time reverse transcription polymerase chain reaction (qRT-PCR) converted to minutes for 143 PEDV N gene-positive clinical samples. The scatter plot was generated in PyCharm 2023.1.3 software by executing function citation (lmplot). Pearson’s correlation coefficient (r) was 0.760 (*p* < 0.001) for intestinal samples and 0.748 (*p* < 0.001) for fecal samples. Shaded areas represent 95% confidence intervals for each sample type.

To confirm the relationships among the detection times for the clinical samples, the results of the clinical evaluation obtained using mqRT-LAMP and qRT-PCR assay were assessed using the Pearson correlation analysis. Pearson’s correlation coefficient (r) for the 85 intestinal and 58 fecal PEDV-positive samples was 0.760 (*p* < 0.001) and 0.748 (*p* < 0.001), respectively. This suggests a significant and strong positive correlation between the capacity of mqRT-LAMP and qRT-PCR to detect the PEDV-N gene in both intestinal and fecal samples. With respect to reaction speed, the mqRT-LAMP assay generated positive signals for intestinal and fecal samples within the range of 7.88–32.14 min (mean: 11.96 ± 4.51 min) and 9.57–30.93 min (mean: 14.52 ± 5.65 min), respectively. In contrast, the qRT-PCR assay required longer reaction times of 36.59–83.12 min (mean: 54.12 ± 11.14 min) and 44.37–91.41 min (average: 62.81 ± 14.23 min), respectively (Figure 3).

## 4. Discussion

In a previous study, a visual RT-LAMP assay using hydroxynaphthol blue metal indicator was developed for field diagnosis of the PEDV virus, which continues to inflict damage on the pig industry worldwide [26]. Here, we combined the LAMP assay, which is a rapid, sensitive, and specific diagnostic method, with an assimilating probe method to enable real-time detection of the virus. To the best of our knowledge, this mqRT-LAMP assay is the first such assay that can simultaneously amplify both the PEDV N gene and EIPC using target-specific probes for the real-time detection of PEDV. This method is more specific and reliable than the previously developed conventional RT-LAMP and real-time RT-LAMP assay which relies on non-target-specific detection methods [22,23,24,25,26,40]. We adopted the assimilating probe method because assimilating probes can be easily designed by incorporating an oligonucleotide complementary to the Q strand into the basic Loop primer sequence. Moreover, the designed F and Q strands can be labeled with fluorescence reporter and quencher dyes at the ends, allowing for straightforward probe design. The analytical sensitivity of the novel probe-based mqRT-LAMP assay was comparable to that of the qRT-PCR assay and proved to be 100-fold higher than that of the RT-PCR assay for PEDV N gene standard RNAs. Furthermore, the precision (repeatability and reproducibility) of the mqRT-LAMP assay for the detection of the PEDV N gene was fully acceptable for a molecular diagnostic assay [41].

As part of our development efforts, we determined that Tp values were highly correlated with copy number across the range of 10^6^ to 10^4^ gene copies (*R*^2^ > 0.98) (Figure 2B,D,F). This suggests that the qRT-LAMP assay described here can be used as a semi-quantitative assay for the detection of PEDV RNA as previously described [28,33,35,42]. However, in the clinical evaluation, the diagnostic sensitivity of mqRT-LAMP was found to be in almost-perfect agreement with quantitative qRT-PCR according to the kappa statistics analysis (Table 4). Additionally, Pearson correlation analysis indicated a significant and strong positive correlation between the mqRT-LAMP and qRT-PCR assays in detecting the PEDV-N gene in clinical samples (Figure 3). These findings suggest that mqRT-LAMP can serve as an alternative to qRT-PCR and can be effectively utilized as a quantitative assay.

To improve the reliability of the developed assay, we used the porcine housekeeping gene, *Sus scrofa β-actin*, as an EIPC that is present in most types of pig samples such as nasal swabs, lung, heart, born marrow, brain, and any other tissue samples [43,44]. In addition, previously reported PCR-based molecular diagnostic assays have adopted the β-actin gene as EIPC and used it to validate fecal samples [45,46]. In this study, we designed a set of LAMP primers and probes to amplify *Sus scrofa β-actin* fragments (Table 1). The performance of *Sus scrofa β-actin* (EIPC) in mqRT-LAMP was evaluated analytically, indicating that EIPC does not interact with the PEDV N gene targets or affect the sensitivity or amplification efficiency of the assay for PEDV detection (Figure 2). The mqRT-LAMP assay successfully detected the *Sus scrofa β-actin* gene when validating specificity using samples that contained porcine-derived material. Moreover, out of the 185 clinical pig samples assessed for clinical evaluation, it generated an EIPC-positive signal for all but eight samples (five fecal and three intestinal samples). This allowed the filtering of samples that were invalid due to inadequate sampling or nucleic acid extraction, ensuring the reliability of the developed mqRT-LAMP assay (Table 4).

Furthermore, the mqRT-LAMP assay amplified PEDV RNA from an additional single qRT-PCR-negative fecal sample, eight RT-PCR-negative fecal, and six RT-PCR-negative intestinal samples that were confirmed as true positive by sequence analysis (Table 4, Supplementary Table S1). Sensitivity analysis using PEDV standard RNA shows that the LOD of mqRT-LAMP was below 5 × 10^1^ copies/reaction and that of RT-PCR was 5 × 10^3^ copies/reaction. The higher sensitivity of mqRT-LAMP may explain the difference in clinical sensitivity with RT-PCR. Furthermore, the single mqRT-LAMP assay-positive and qRT-PCR-negative discrepant fecal sample (KNU_56) with clearly detected EIPC showed signals of high mqRT-LAMP Tp values (Tp of 32.33). These discrepancies may be due to the presence of unknown inhibitors in the fecal samples that are specific to the qRT-PCR assay, or the differences in clinical diagnostic sensitivity between the two diagnostic assays [18,19]. Fecal and intestinal samples are known to contain inhibitors such as bilirubin and bile salts, which can negatively affect the sensitivity of RT-PCR [47,48]. However, LAMP assay can detect trace amounts of a target gene more effectively while maintaining comparable analytical sensitivity, because LAMP assay using *Bst* DNA polymerase is less sensitive to inhibitors in a clinical sample solution than PCR assay using Taq DNA polymerase [49,50].

The LAMP diagnostic method, which conducts reactions under isothermal conditions, provides a rapid diagnosis compared to PCR or real-time RT-PCR, while not mandatorily requiring expensive instrumentation. It also has the advantage of being applicable in the field with minimal equipment. The assimilating probe strategy adopted in this study could be useful for converting known conventional LAMP assays already in use for the detection of other pathogens into real-time monitoring systems. The newly developed probe-based mqRT-LAMP can identify specific amplifications of target genes in real time without waiting until the endpoint of the response. Both sensitivity and specificity were similar to those of qRT-PCR used in the previously reported PEDV diagnostic method and significantly reduced the reaction time from 2 h to within 30 min.

However, our study is subject to the limitation that all experimental data were obtained using relatively costly real-time PCR equipment, rather than utilizing more affordable portable systems such as Biomeme Franklin™ three9 Real-Time PCR Thermocycler (Biomeme, Philadelphia, PA, USA) or Genie III (Optigene Ltd., Horsham, UK). Additionally, the experiment was conducted in a well-equipped laboratory using highly purified and efficiently extracted nucleic acids, which may not fully mirror the complexities and constraints encountered in field applications. To utilize the assay for on-site diagnosis, further study is therefore needed to verify that the newly developed mqRT-LAMP assay can be properly performed using even relatively simple devices and nucleic acid extraction.

In conclusion, the developed mqRT-LAMP assay exhibited exceptional sensitivity and specificity in detecting PEDV RNA, and the inclusion of the EIPC further bolstered the assay’s reliability. The approach will be of great help in the rapid diagnosis and prevention of transmission of PEDV and is likely to have applications beyond well-equipped laboratories, rendering it a highly valuable diagnostic tool in resource-limited settings or developing countries.

## Figures and Tables

**Table 1:** Primers and probes used in this study.

Assay	Primer and Probe	Sequence (5′-3′)	Target Gene	Genome Position ^a^	Reference
mqRT -LAMP	F3	CTTCGAARGAACGTGACCT	PEDV *N*	27,184–27,202	[26] & this study
	B3	CAATGCTGCAACATTTGGT		27,356–27,374	
	LF	GCTATTTTCGCCCTTGGGA		27,230–27,248	
	LB	AGGTGTTGATGCSTCAGG		27,314–27,331	
	FIP (F1c + F2)	TGGGTCCGAAGCAAGCTG+ AGACATCCCAGAGTGGAGG		27,253–27,270 + 27,206–27,224	
	BIP (B1c + B2)	TTGGAGATGCGGAATTTGTCG+ AACTGGCGATCTGAGCATAG		27,289–27,309 + 27,332–27,351	
	PEDV-LF-F	FAM-ATAAGGTCCTCGCCGCTCAAGATAGGCAGA- GCTATTTTCGCCCTTGGGA			
	Q	TCTGCCTATCTTGAGCGGCGAGGACCTTAT-BHQ1			
	EIPC-F3	CATCCTGCGTCTGGACCT	*Sus scrofa* *β-actin*	609–626	This study
	EIPC-B3	AGCTCTTCTCCAGGGAGG		788–805	
	EIPC-LF	CGCTCCGTCAGGATCTTCAT		655–674	
	EIPC-LB	CTACGTCGCCCTGGACTTC		738–756	
	EIPC-FIP (F1c + F2)	CCGTGGTGGTGAAGCTGTAGC+ GGACCTGACCGACTACCTC		677–679 + 636–654	
	EIPC-BIP (B1c + B2)	AGATCGTGCGGGACATCAAGG+ AGTGGCCATCTCCTGCTC		707–727 + 757–774	
	EIPC-LF-F	HEX-ATAAGGTCCTCGCCGCTCAAGATAGGCAGA- CGCTCCGTCAGGATCTTCAT			
	Q	TCTGCCTATCTTGAGCGGCGAGGACCTTAT-BHQ1			
RT- PCR	P1	TTCCCAGCGTAGTTGAGATTG	PEDV *N*	26,761–26,781	[25]
	P2	CGAAGTGGCTCTGGATTTGTT		27,168–27,188	
qRT- PCR	NF	CGCAAAGACTGAACCCACTAA	PEDV *N*	26,684–26,704	[13] modified
	NR	TTGCCTCTGTTGTTACTTGGAGAT		26,858–26,881	
	NP-FAM	FAM–TGTTGCCATTRCCACGACTCCTGC–BHQ1		26,824–26,847	

^a^ Genome position of primers and probe sequences according to Korean representative PEDV genome sequence (GenBank accession No. KR873431), and EIPC sequences according to the *Sus scrofa β-actin* genome sequence (GenBank accession NO. AY550069). The underlined text represents the LF primer sequence used to design the assimilating probe.

**Table 2:** Specificity of mqRT-LAMP using PEDV and EIPC-specific primer and probe sets.

Pathogen	Strain	Source ^a^	Amplification of Target Gene (Tp)	
			PEDV N Gene (FAM)	EIPC ^b^ (HEX)
Porcine epidemic diarrhea virus	KNU141112-S DEL5/ORF3	CVAS	9.69	−
Transmissible gastroenteritis virus	175Lvac	APQA	−	15.8
Porcine delta coronavirus	KNU16-07	IVBS	−	16.6
Porcine rotavirus	A1Va	IVBS	−	−
Porcine circovirus type 2	PCK0201	IVBS	−	11.2
Porcine reproductive and respiratory syndrome virus type 1	Lelystad	APQA	−	−
Porcine reproductive and respiratory syndrome virus type 2	LMY	APQA	−	−
Classical swine fever virus	LOM	APQA	−	21.8
Swine influenza virus	VDS1	IVBS	−	−
Porcine parvovirus	NADL-2	IVBS	−	14
Aujeszky’s disease virus	YS	IVBS	−	12.7
Non-infected pig fecal sample	−	IVBS	−	13.7
Non-infected pig intestinal sample	−	IVBS	−	11.4
PK-15 cell	−	IVBS	−	10.9
Vero cell	−	IVBS	−	−

^a^ CAVS, commercially available vaccine strain; IVBS, Institute for Veterinary Biomedical Science (Kyungpook National University, Republic of Korea); APQA, Animal and Plant Quarantine Agency (Gimcheon, Republic of Korea); EIPC, endogenous internal positive control. −: negative reaction. ^b^ HEX fluorescence signals for porcine EIPC were obtained from porcine pathogens and cells of porcine origin.

**Table 3:** Precision of multiplex real-time reverse transcription loop-mediated isothermal amplification assay (mqRT-LAMP).

Dilution (Copies/µL)	Intra-Assay Tp Value (min)			Inter-Assay Tp Value (min)		
	High (10^6^)	Medium (10^4^)	Low (10^2^)	High (10^6^)	Medium (10^4^)	Low (10^2^)
Mean	8.73	10.47	15.04	9.09	11.20	17.45
SD	0.23	0.39	0.49	0.09	0.09	0.18
CV (%)	2.65	3.76	3.28	0.95	1.74	1.01

The mean value (mean), standard deviation (SD), and coefficient of variation (CV) were determined based on the time to positive (Tp) values by mqRT-LAMP using three different concentrations of standard RNAs of PED nucleocapsid (N) genes.

**Table 4:** Comparison of mqRT-LAMP, qRT-PCR, and RT-PCR results for the detection of porcine epidemic diarrhea virus in clinical samples.

Method		mqRT-LAMP Assay			Positive Rate	Overall Agreement (%)
		Positive	Negative ^a^	Total		
qRT-PCR	Positive	142	0	142	76.8%	99.5%
	Negative	1	42	43		
	Total	143	42	185		
RT-PCR	Positive	129	0	129	69.7%	92.4%
	Negative	14	42	56		
	Total	143	42	185		
Positive rate		77.3%				

The calculated kappa coefficient value (95% confidence interval) between multiplex real-time reverse transcription loop-mediated isothermal amplification (mqRT-LAMP) and real-time reverse transcription polymerase chain reaction (qRT-PCR) or reverse transcription polymerase chain reaction (RT-PCR) was 0.98 (0.95–1.02) and 0.80 (0.71–0.91), respectively. ^a^ HEX signals for EIPCs (*Sus scrofa β-actin* gene) were not generated in five fecal and three intestinal clinical samples.

## Data Availability

Not applicable.

## Supplementary Materials

The following supporting information can be downloaded at: https://www.mdpi.com/article/10.3390/v15112204/s1. Figure S1: Sensitivities of real-time reverse transcription-polymerase chain reaction (qRT-PCR) and reverse transcription-polymerase chain reaction (RT-PCR) assay previously reported in other studies. Table S1: Comparison of diagnostic results for 14 discrepant clinical samples between the newly developed multiplex real-time reverse transcription loop-mediated isothermal amplification (mqRT-LAMP) assay and previously reported qRT-PCR and RT-PCR assays.
