# Supplementary material for: An Advanced Multiplex Real-Time Reverse Transcription Loop-Mediated Isothermal Amplification Assay for Rapid and Reliable Detection of Porcine Epidemic Diarrhea Virus and Porcine Internal Positive Control
Source: Viruses. 2023 Nov 1;15(11):2204. doi: 10.3390/v15112204 (PMC10674262; doi:10.3390/v15112204)
Supplement: Supplementary file 1 [file viruses-15-02204-s001.zip › viruses-2686422-supplementary.pdf]

Supplementary Material

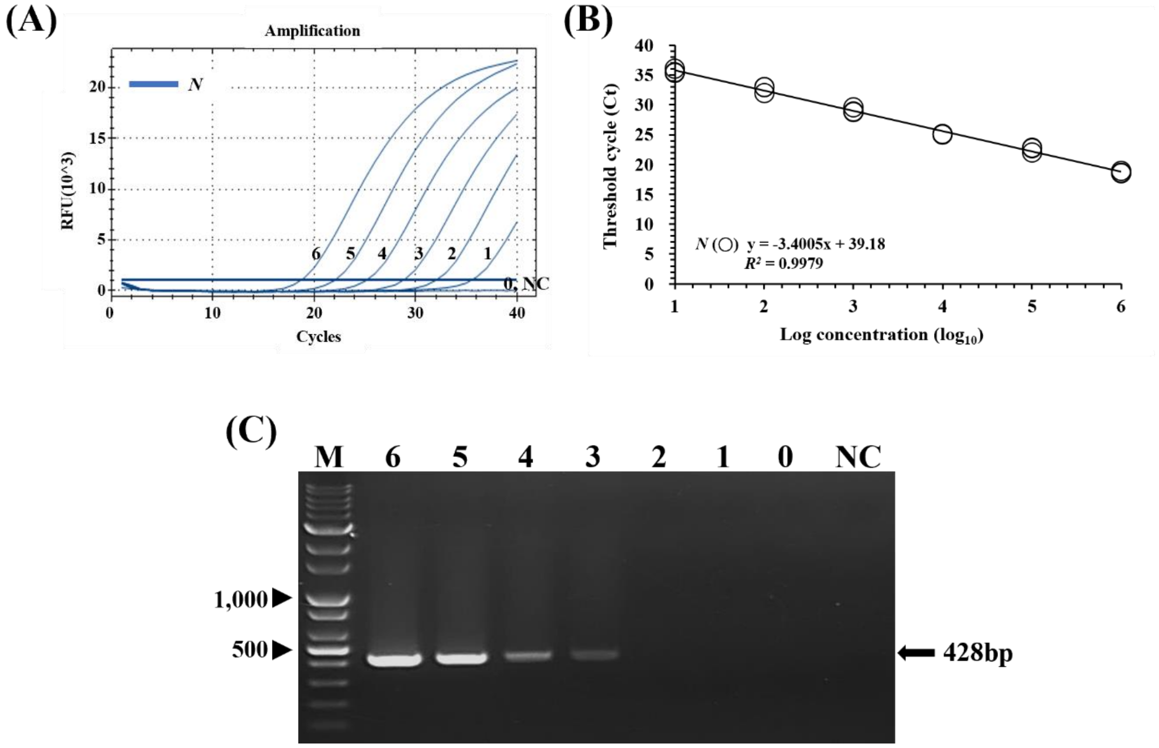

Figure S1. Sensitivities of real-time reverse transcription-polymerase chain reaction (qRT-PCR) and reverse transcription-polymerase chain reaction (RT-PCR) assay previously reported in other studies. (A, B) Limit of detection (LOD) of qRT-PCR assay and standard curve. (C) Electrophoretic analysis of RT-PCR amplified products. Amplification curve and electrophoretic analysis are shown using one of three replicates. Lines 6–0 show a 10-fold serial dilution of RNAs (from 10^6 to 1 copies/μL). Lane M, 1 kb plus DNA ladder. NC, negative controls (nuclease-free water).

Table S1. Comparison of diagnostic results for 14 discrepant clinical samples between the newly developed multiplex real-time reverse transcription loop-mediated isothermal amplification (mqRT-LAMP) assay and previously reported qRT-PCR and RT-PCR assays.

NO	Sample code	Sample type	Results of different assays			
			mqRT-LAMP (Tp value)		qRT-PCR (Ct value)	RT-PCR
			PEDV N gene	Sus scrofa β-actin gene	PEDV N gene	PEDV N gene
1	KNU_25	Feces	25.41	15.74	33.38	-
2	KNU_27	Feces	21.80	19.48	34.34	-
3	KNU_56	Feces	32.33	11.50	-	-
4	KNU_66	Feces	30.13	13.50	35.40	-
5	KNU_67	Feces	12.92	17.55	36.55	-
6	KNU_95	Feces	27.77	28.49	33.04	-
7	KNU_96	Feces	22.15	20.82	33.72	-
8	KNU_97	Feces	22.36	26.77	33.05	-
9	KNU_154	Intestine	32.14	11.94	31.74	-
10	KNU_158	Intestine	9.35	9.94	15.41	-
11	KNU_162	Intestine	24.32	10.08	29.11	-
12	KNU_163	Intestine	26.23	11.02	32.58	-
13	KNU_165	Intestine	28.72	10.00	32.78	-
14	KNU_183	Intestine	13.75	10.62	23.73	-

'-', negative result.
